# Diagnostic and therapeutic delay in Rheumatoid Arthritis patients: Impact on disease outcome

**DOI:** 10.12669/pjms.37.4.3471

**Published:** 2021

**Authors:** Faiza Naeem, Saira Elaine Anwer Khan, Muhammad Ahmed Saeed, Sumaira Farman

**Affiliations:** 1 Faiza Naeem, FCPS Internal Medicine, Rheumatology Fellow, Division of Rheumatology, Fatima Memorial Hospital College of Medicine & Dentistry (FMH), Lahore, Pakistan; 2 Saira E.A Khan, MRCP (UK), SCE Rheumatology (UK) Assistant Professor, Division of Rheumatology, Fatima Memorial Hospital College of Medicine & Dentistry (FMH), Lahore, Pakistan; 3 Muhammad Ahmed Saeed, FCPS Rheumatology, FACR, FCPS Internal Medicine Associate Professor, Al-Aleem Medical College, Head Department of Rheumatology, Gulab Devi Teaching Hospital, Lahore, Pakistan. Consultant Rheumatologist, National Hospital and Medical Center, Lahore; 4 Sumaira Farman, FRCP, FACP, FACR, SCE Rheumatology (UK) Graduate Certificate Paediatric Rheumatology (Australia) Professor, Division of Rheumatology, FMH, Consultant Rheumatologist, National Hospital and Medical Center, Lahore, Pakistan

**Keywords:** DAS28, Diagnostic delay, HAQ-DI, Rheumatoid arthritis, Therapeutic delay

## Abstract

**Objective:**

To identify factors causing diagnostic and therapeutic delay in patients with rheumatoid arthritis, and to evaluate relationship of diagnostic and therapeutic delay with disease outcome.

**Methods:**

This cross-sectional study was conducted in Rheumatology Department, Fatima Memorial Hospital, Lahore, Pakistan, from May 2018 to July 2018. In this study 102 patients fulfilling ACR/EULAR criteria 2010 were enrolled. Lag times were calculated in months: lag-1 (delay in initial medical consultation); lag-2 (delay in consulting rheumatologists); lag-3 (diagnostic delay); lag-4 (therapeutic delay). Disease activity and functional outcome were measured by DAS28, HAQ-DI respectively. Association of lag-3 and lag-4 with HAQ-DI and DAS28 was calculated by Pearson correlation.

**Results:**

Median (IQR) disease duration of study group was 6(2-10) years. Initial consultations were with; orthopedic surgeon 40(39.2%), general practitioner 27(26.5%), rheumatologist 13(12.7%), medical specialists 14(13.7%). Median (IQR) lag times in months: lag-1 (delayed initial consultation): 2(0-5), lag-2 (delay in consulting rheumatologist): 30(7.7-72), lag-3 (diagnostic delay): 12(3-48), lag-4 (therapeutic delay):18(5.7-72). Factors attributed to lag-3 (diagnostic delay) and lag-4 (therapeutic delay) (p<0.05): older Age (r= 0.2), education level(r= - 0.2), initial consultation (non-rheumatologist) (r=0.2), lag-2(r=0.8), >three doctors visited before diagnosis(r=0.6). Positive anti-CCP antibodies(r=0.2) and lag-1 (delayed initial consultation) (r=1) were associated with lag-3 (diagnostic delay) only; no association was found with positive RA factor. Significant correlation (p=<0.05) of lag-3 (diagnostic delay) was found with both DAS28(r=0.2) & HAQ-DI(r=0.2). Similarly lag-4 (therapeutic delay) also correlated with both & DAS28(r=0.2) & HAQ-DI(r=0.3) (p=<0.05).

**Conclusion:**

Diagnostic and therapeutic delay were associated with older age, lower education and delayed consultation with rheumatologist but not with positive RA factor. Positive anti-CCP antibodies were associated with diagnostic delay only. Diagnostic and therapeutic delay led to high disease activity and poor functional outcome in RA patients.

## INTRODUCTION

Rheumatoid arthritis (RA) is an autoimmune, systemic, chronic inflammatory disorder with articular and extra-articular manifestations.^1^ Worldwide RA affects 0.24 to 1% adult population^2^ with female to male ratio 3:1. Peek age of onset in females is late childbearing years while in males is in sixth to seventh decade.^1^ Point prevalence of RA reported from Karachi has increased from 12.9% in 2011 to 26.9% in 2015.^3^ Musculoskeletal disorders contribute to 6.7% of “total overall global burden” of disease.^4^

Damage is irreversible once erosions develop on radiograph leading to deformities within one to two years.^1^ In 2013 average cost of illness of RA patient in South India was estimated as 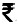
2229.99 ($34)/month.^5^ Regarding health economics, long term studies reported that 50% RA patients have had to stop working after 10 years of disease which is 10-times the average rate caused by other medical conditions.^1^ In RA the overall mortality is 2.5 times higher than that of the general age-matched population.^6^ RA is associated with fatty liver and comorbidities like metabolic syndrome, diabetes mellitus and hypertension.^7^ Hypertension, smoking and self-use NSAIDS/Hakeem medication lead to impaired renal function in RA patients.^8^

Initiating treatment within 12 weeks of symptoms onset (therapeutic window of opportunity)^9^ doubles the chances of achieving remission and decreases necessity of using biologics in RA treatment from 32.24% to 10%^10^,^11^ leading to better functional outcome (lower HAQ-DI values) with good radiographic outcome and lower disease progression rate.^9^,^12^

Main objective of this study was to identify factors contributing to diagnostic and therapeutic delay in RA patients and to evaluate relationship of diagnostic and therapeutic delay with disease outcome, previously never studied in Pakistan. Considering the data available on disease burden and disability^2^,^3^,^6^ as well as economic burden caused by RA,^5^ it is need of the hour to address these factors to improve outcome of RA in Pakistani milieu. Delays in diagnosis and initiation of treatment for Rheumatoid arthritis leads to poor disease outcome in terms of disability and quality of life, early diagnosis and treatment will result in improved quality of life and prevention of complications of Rheumatoid arthritis.

## METHODS

This cross-sectional study was conducted in Rheumatology department, Fatima Memorial Hospital, Lahore, Pakistan, from May 2018 to July 2018. After receiving approval from Hospital Ethical Committee (dated June 01, 2018, IRB FMH-O4-2018-IRB-419-M), total one hundred and two patients, both male and female with RA fulfilling ACR/EULAR^(annex-1)^ classification criteria 2010, with age ≥18 years, and disease duration of ≥3 months were included using 95% Confidence level (SD=1.96), 10% margin of error (SE=0.1), keeping 31% patients (P=0.31) assessed within 12 weeks, showed less joint destruction and higher chances of remission,^9^ Q=1-P, using following formula:


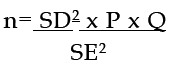


### Exclusion criteria:

- RA overlapping other connective tissue diseases.
- Comorbidity/medical illness leading to functional impairment.
- Pregnancy.

### Operational definitions:

Diagnostic and treatment delay was defined as delay in establishing RA diagnosis and initiating treatment after 12 weeks (therapeutic window of opportunity)^9^ and was calculated in lag times.^13^

- **Lag-1:** delay in initial consultation from symptoms onset.
- **Lag-2:** delay in consulting rheumatologist from symptoms onset.
- **Lag-3 (diagnostic delay):** delay in diagnosis from symptoms onset.
- **Lag-4 (therapeutic delay):** delay in initiating 1st DMARD from symptoms onset.

Impact on disease outcome was measured by calculating disease activity and functional disability; DAS28 ^(annex2)^ and HAQ-DI ^(annex3,4,5)^ respectively. Threshold of disease activity with DAS28 was interpreted as follow: Remission <2.6, low activity >2.6-<3.2, moderate activity ≥3.2-≤5.1, high activity >5.1.^14^ HAQ-DI index was interpreted in three categories: 0-1: mild to moderate disability, 1-2: moderate to severe disability, 2-3: severe to very severe disability.^15^

Data was analyzed by SPSS version 17 and quantitative study variables like age were presented as mean and standard deviation. Qualitative variables like gender and factors were presented as frequency and percentages. Lag time1-4 and disease duration were calculated in median and interquartile range (IQR). All patients were divided into three groups according to diagnostic and therapeutic delay (lag-3 & lag-4) of ≤1 year, >1-≤5 years and >5 years. Mean DAS28 & HAQ-DI values were calculated for each group and were compared among them by applying ANNOVA. Lag-3 and lag-4 were correlated with both DAS28 and HAQ -DI by using Pearson correlation. Spearman correlation was used to correlate all factors with both lag3 and lag4. P<0.05 was taken as significant.

## RESULTS

### Initial consultation

Initial consultations were with; orthopaedic surgeon 40 (39.2%), general practitioner 27 (26.5%), rheumatologist 13(12.7%), medical specialists 14 (13.7%), hakeem 7(6.9%), homeopathic 1 (1%). Lack of awareness (50.5% of patients) and lack of referral (49.55% of patients) were causes of delay in consulting rheumatologists. At initial consultation RA was diagnosed in only 49 (48%) patients. Rheumatologists diagnosed RA in 48(47.1%) patients, orthopaedic surgeons in 30(29.4%), medical consultants in 15(14.7%) and GP in 9(8.9%) patients. Rheumatologists started 1^st^ DMARDS in 78(76.5%) and non-rheumatologists in 24(23.5%) patients.

### Lag times

Median (IQR) lag times were calculated in months.

- **Lag-1**: 2(0-5).
- **Lag-2:** 30(7.7-72).
- **Lag-3:** 12(3-48).
- **Lag-4:** 18(5.7-72).

### Diagnostic & therapeutic delay (Lag-3 & Lag-4) with disease outcome

Patients having diagnostic and therapeutic delay of one year or less were found to have lower disease activity and better functional outcome. (Table-III).

## DISCUSSION

In our study group, lag-1 (delay in initial consultation) was two (0-5) months, shorter than that (3.1(0-5.7) months) observed in developed countries like Canada, USA, UK.^13^ However, lag-1 in Saudi Arabia^16^ was six months.

This study showed that median lag-3 (diagnostic delay) was one year while median lag-4 (therapeutic delay) was one and half years but initial consultations were done within “window period of opportunity”. On average >three physicians were consulted before RA diagnosis was reached (Table-I). Median lag-2 (delay in consulting rheumatologists) was 30(7.7-72) months. Rheumatologists were ultimately responsible for diagnosing RA.

**Table-I:** Demographic data of patients in the study.

Characteristics	All patients (n=102)
Age (years)	40.9±11.9
Female	93(91.2%)
Rural	33(32.4%)
Urban	65(63.7%)
***Qualification:***	
• None	23(22.5%)
• Read and write only	4(3.9%)
• Primary education	22(19.6%)
• Secondary education	29(28.4%)
• Degree	25(24.5%)
Disease duration: median (IQR)	6(2-10) years
RA factor positive	80(78.4%)
Anti-CCP antibodies	68(66.7%)
***Pattern of joints involvement at onset:***	
• Hand and wrist	58(56.9%)
Bilateral/unilateral	41.4%/15.2%
• Knee	43(42.2%)
Bilateral/unilateral	42.25%/17.6%
•Foot and ankle	18(17.6%)
Bilateral/unilateral	17.65/3.9%
Average no. of doctors consulted before diagnosis	3.68±3.1

Data presented as means & SD, and frequency and percentage and disease duration was calculated in median, interquartile range (IQR).

Median (IQR) time in months for lag-2: 30(7.7-72); lag-3: 12(3-48) & lag-4: 18(5.7-72) were greater than those observed in developed countries like Canada, USA, UK where measured median (IQR) lag-2 was 2.13(0.5-6.6) months, lag-3 was 2.91(0-5) months, lag-4 was 2.14(0-2.2) months.^13^ In contrast, diagnostic delay in Kingdom of Saudi Arabia was about 30 months.^16^ In Maharashtra (India), median (IQR) lag time to consult rheumatologist was 24(1-372) months.^17^ These lag times were not measured previously in any local study from Pakistan.

Initial consultation with rheumatologists was done by 12.7% patients only. Major causes of delay in seeking initial consultation with rheumatologist were lack of awareness and lack of referral. One study conducted in Rawalpindi reported that even in diagnosed RA patients only 1.5% patients had knowledge on disease.^18^ Current study shows only 47.1% of cases were diagnosed by rheumatologist hence this lack of initial rheumatologist consultation led to lag-3(r=0.2, p=0.04) and lag-4(r=0.29, p=0.08) (Table-II). This was in contrast to study by Hussain W et al, where rheumatologists diagnosed RA in 75.6% patients.^16^ Even though non-rheumatologists diagnosed RA, they did not initiate DMARD’s. Initiation of DMARD’s in majority of cases was done by rheumatologists 78(76.5%). Longer the delay in initial consultation with rheumatologists, longer were lag-3(r=0.47, p=0.00) and lag-4(r=0.29, p=0.00) (Table-II). Causes of diagnostic and therapeutic delay were delayed initial consultation, primary care physicians (lack of experience in recognizing RA and in use of DMARD therapy), delayed referral^19^ and use of NSAIDS (which mask RA symptoms).^20^ Greater the number of doctors visited before diagnosis, longer were the diagnostic and therapeutic delay (r=0.20, p=0.04 & r=0.29, p=0.002) (Table-II). Similar pattern was reported from Saudi Arabia (p=<0.00).^16^

**Table-II:** Factors causing diagnostic and therapeutic delay in RA

Factors	Lag-3 Median (IQR) months	p-value	Lag-4 Median (IQR) months	p- value	Association with lag-3	Association with lag-4
***Age***						
18-30 years	7(21)	>0.05	12(43)	>0.05	r=0.22	r=0.21
30-50 years	12(53.8)		21(66.3)		p=0.02	p=0.03
>50 years	36(69)		48(114)			
***Gender***						
Female	12(33)	>0.05	18(57)	>0.05	r=0.06	r=0.02
Male	24(104.5)		38(104.5)		p=0.51	p=0.76
***Qualification***						
Illiterate - read & write only	24(54)	>0.05	36(60)	<0.05	r=- 0.20	r=- 0.29
Primary	18(69)		48(6)		p=0.04	p=0.03
Secondary and above	7(30)		12(35)			
Rural	12(57)	>0.05	24(79)	>0.05	r=0.001	r=0.04
Urban	12(33)		18(54.5)		p=0.99	p=0.66
***Hand & wrist***						
Yes	8(48)	>0.05	18(79)	>0.05	r=- 0.035	r=- 0.01
No	12(36)		21(53.8)		p=0.72	P=0.91
***Knee joint***						
Yes	8(30)	>0.05	12(42)	>0.05	r=0.13	r=0.06
No	12(56.8)		24(66.3)		p=0.18	p=0.52
***RAF positive***						
Yes	9(33)	>0.05	18(57.5)	>0.05	r=0.14	r=0.06
No	42(66)		48(66)		p=0.16	p=0.94
***Anti-CCP positive***						
Yes	8(33)	<0.05	15(62.8)	>0.05	r=0.20	r=0.07
No	36(65)		36(65)		p=0.03	p=0.43
***Initial consultation***						
• Non rheumatologist	12(50.5)	<0.05	24(65.5)	<0.05	r=0.20	r=0.29
• Rheumatologist	6(8.5)		6(8.5)		p=0.04	p=0.002
***Lag-1***						
• 0-≤6 months	7.5(33)	<0.05	12(55.3)	<0.05	r=0.25	r=0.17
• >6 months-<1 years	12(70)		36(70)		p=0.01	p=0.08
• >1year	48(112)		48(112)	
***Lag-2***						
• ≤1 years	4.5(5.5)	<0.05	5.5(90)	<0.05	r=0.47	r=0.76
• >1 year-<5 years	24(41)		24(41)		p=0.00	p=0.00
• ≥5 years	72(119.5)		108(66)	
***Average no of doctors visited before diagnosis***						
• <3	6(18)	<0.05	7(20.5)	<0.05	r=0.46	r=0.60
• >3	36(70)		72(66)		p=0.00	p=0.00

Mann-Whitney U test or Kruskal-Wallis H test was used to compare median lag-3 and median lag-4 for each factor (p<0.05 significant) to reflect whether median lag-3 & lag-4 were statistically different between males and females, between RAF**^+^** (rheumatoid arthritis factor) & RAF^⁃^ patients, between anti-CCP**^+^** antibodies (anti-cyclic citrullinated peptide) and anti-CCP^⁃^ antibodies patients, and lag-1 and lag-2 etc. Spearman correlation was used to correlate lag-3 and lag-4 with factors (p value <0.05 significant).

Gender and geographical region (rural vs. urban), presence of initial symptoms (hand/wrist, knees involvement) were not found to be associated with lag-3 and lag-4. Delay in initial consultation led to diagnostic delay (r=0.25, p=0.01). Older age was associated with lag-3 (r=0.2, p=0.02) and lag-4 (r=0.21, p=0.03) (Table-II). Hussain W et al, reported that patients presenting initially with hand/wrist involvement and fatigue as well as those belonging to urban population were associated with early diagnosis but no association of diagnostic delay was found with gender and age.^16^ Both lag-3 and lag-4 correlated with lower education status (r=-0.20, p=0.04 &r=-0.29, p=0.03 respectively) (Table-II). Cho et al, reported that older age at onset, higher education level and higher income led to early diagnosis.^21^

We did not find any association of RA factor+/- with diagnostic and therapeutic delay. Positive anti-CCP antibodies were associated with diagnostic delay (r=0.20, p=0.03) but not with therapeutic delay (r=0.07, p=0.43) (Table-II). Pratt et al, described that insidious onset of symptoms in patients with RA factor^+^/anti-CCP^+^ led to delay in seeking medical care which in turn led to diagnostic delay. Diagnostic and therapeutic delay in RA factor^-^/anti-CCP^-^ patients occurred in secondary care.^22^ One study conducted in Karachi reported that RA factor- patients had diagnostic delay and hence delayed rheumatology referral.^23^ All the other possible factor causing diagnostic and therapeutic delay were not determined previously in Pakistan as we have tried to find out to reduce lag times in order to improve disease outcome in RA patients.

**Fig.1:**
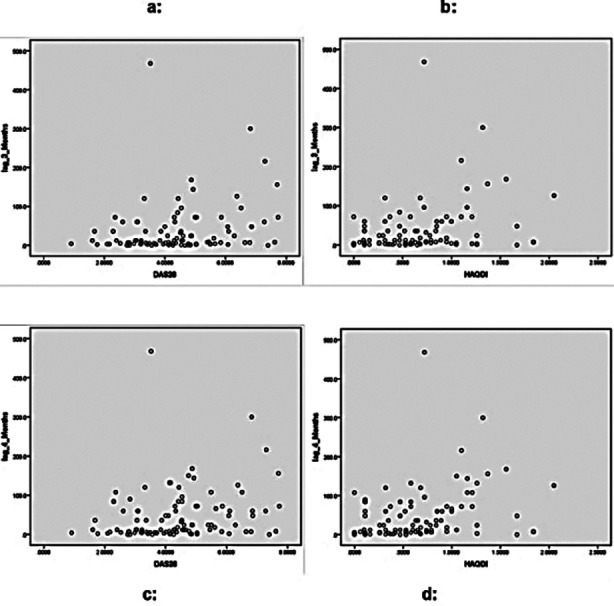
Association of Lag-3 and Lag-4 with DAS28 and HAQ-D1 (Impact on disease outcome) Pearson correlation was used to correlate lag-3 and lag-4 with HAQ-D1. Significant correlation between lag 3 (diagnostic delay) and DAS28 was found (r=0.2, p=0.02) (Fig. a) as well as between lag3 and HAQ (r=0.2, p= 0.003), (Fig. b) Similarly significant correlation between leg 4 (therapeutic delay) and DAS28 (r=0.2, p=0.03) (Fig. c) as well as between lag4 and HAQ (r=0.3, P=0.001) was found (Fig. d).

Significant statistical correlation of lag-3 was found with both DAS28 (r=0.2, p=0.02), and HAQ-DI (r=0.2, p=0.003) (Fig. 1a & 1b). Correlation of lag-4 with both DAS28 (r=0.2, p=0.03) and HAQ-DI (r=0.3, p=0.001) was found (Fig. 1c & 1d). Badsha et al, reported high disease activity (DAS28) in patients with diagnostic delay (r=0.323, p=0.025).^20^ Kim et al,^12^ reported higher HAQ-DI score in delayed diagnosis group (0.70±0.66) vs. in early diagnosis group (0.6±0.63) p<0.01. We revealed that delay in diagnosis leads to higher mean HAQ-DI and DAS28 scores. Mean HAQ-DI and DAS28 scores were lower in delayed diagnosis group-1; (4.04±1.37 & 0.55±0.44) vs. higher values in group-2; (4.58±1.5 & 0.61±0.40) and group-3; (5.01±1.65 & 0.87±0.48) p=<0.05) (Table-III). Similarly, greater delay in initiating treatment lead to worse HAQ-DI and mean DAS28 values which were lower in group-1; (3.9±1.42 & 0.499±0.44) vs. higher values in group-2; (4.5±1.32 & 0.66±0.38) and group-3; (4.9±1.57 & 0.82±0.46 respectively) p=<0.05 (Table-III). Ragab et al,^24^ reported lower HAQ-DI and DAS28 value in patients having started treatment within six months of symptom onset than those starting treatment later(p=0.01).

**Table-III:** Diagnostic & therapeutic delay (Lag-3 & Lag-4) with disease outcome.

	Groups	Lag time	N%	DAS28 Mean ±SD	P-value	HAQ-DI Mean ±SD	P-value
Lag-3	Group 1	≤1 year	60(58.8%)	4.04±1.37	0.02	0.55±0.44	0.01
	Group 2	>1 year-<5 years	19(18.6%)	4.58±1.5		0.61±0.40	
	Group 3	>5 years	23(22.5%)	5.01±1.65		0.87±0.48	
Lag-4	Group 1	≤1 year	50(49%)	3.9±1.42	0.01	0.499±0.44	0.00
	Group 2	>1 year-<5 years	19(18.6%)	4.5±1.32		0.66±0.38	
	Group 3	>5 years	33 (32.4)	4.9±1.57		0.82±0.46	

Annova was used to compare mean DAS28 & HAQ-DI between groups (group1-group3) for both lag3 and lag4 (p value<0.05 significant).

### Strength and limitations

This single concise study gives true insight into the lag times along with different factors causing diagnostic and therapeutic delay in RA patients and their impact on disease outcome in Pakistan. This study is first of its kind ever done in Pakistan.

### Limitations of the study

All patients in this study belonged to lower socioeconomic status. Other factors e.g. myths and fears of patients regarding disease and treatment, adherence to treatment, and reason of delay in patient with better socioeconomic status were not evaluated in this study. This study needs to be repeated after few years in same population to evaluate any decline in lag times and improvement in disease outcome.

## CONCLUSION

Older age, lower education and delayed consultation with rheumatologist were the factors associated with both diagnostic and therapeutic delay. Positive RA factor did not impact on diagnostic and therapeutic delay. Positive anti-CCP antibodies were associated with diagnostic delay only. Diagnostic and therapeutic delay ultimately led to high disease activity and poor functional outcome in RA patients. It is essential to address these factors to minimise diagnostic and therapeutic delay in RA patients to improve their physical wellbeing and to decrease burden of disease, disability and economic burden in our patients.

### Author’s Contribution:

**FN and SEAK** conceived and designed manuscript.

**FN** collected data, did statistical analysis, wrote & edited manuscript, she is also responsible for integrity and accuracy of research work.

**MAS and SF** reviewed, gave final approval of manuscript.
